# Household Hints and Recipes

**Published:** 1879-07

**Authors:** 


### Household Hints & Recipes.

WAFFLES.—One quart milk, half cup melted butter, yolks of three eggs well beaten, one heaping teaspoonful of baking powder. Beat in flour enough to make a thin batter, and add the well beaten whites of the three eggs the last thing.

---

TO COLOR RED THE EDGES OF BOOKS.—When the edges are trimmed, keep the book in the press and brush on a coating of dilute gum tragacanth (about ½ lb. gum to 1½ gallons), colored to the desired hue with a mixture of 3 parts rose pink with 1 vermillion. Let dry in the press and burnish with an agate burnisher.

---

LIQUID GLUE.—This article is common and useful, can be made easily by diluting officinal phosphoric acid with two parts, by weight of water, and saturating with carbonate of ammonia; dilute the resulting liquid, which must be still somewhat acid, with another part of distilled water, warm it on a water-bath, and dissolve it in enough good glue to form a thick, syrupy liquid. It must be kept in well closed bottles.

---

SAWDUST IN ART.—In Germany, according to the *Polytechnic Review*, sawdust is employed in the production of sundry articles, both useful and ornamental. A plastic mass is prepared, composed two-thirds of hard-wood sawdust and one-third glue, resin, or other binding material. This is compressed in brass moulds, and the moisture driven out by heat. The articles made are bass-reliefs, piano-keys, door-knobs, brush handles and backs, etc.

---

MOTH DESTROYER.—In the November number of the *Druggists' Circular* a paragraph on this subject was quoted from the *American Agriculturist*, in which petroleum was recommended for this purpose. It ought to be mentioned that when benzine, naptha, or any such volatile and explosive matters are employed, flame and fire are to be kept carefully away, or there may be unexpectedly a conflagration. The remedy is no doubt thorough, but its use requires caution. In this city there are one or more quite extensive concerns which make a business of purging away all sorts of vermin that infest upholstered work and bedding by using benzine.

CORN BREAD.—Beat two eggs, white and yolks separately, one pint of sour milk or buttermilk, two tablespoonfuls sugar, one tablespoonful butter, melted, but not hot, a little salt. Mix these all but the whites of the eggs. Reserve them for the last. Put two-thirds of a teaspoonful of soda, rolled perfectly free from lumps, into a pint of corn meal and sift both together, then stir into the milk, eggs, etc., beat well and add the whites of the eggs the last thing. Put into a well-buttered pan and bake.

---

MOTHS IN FURNITURE.—Taken as a whole writes the *American Agriculturist*, throughout the country, housekeepers suffer more annoyance and destruction of furniture, carpets and woolen garments by moths than from any other pest. The little red ant is a great nuisance in some localities, but it is not destructive, and not very prevalent. Moths are universal, and whole sets of costly upholstered furniture fade away, losing their beauty and substance, even after days, weeks, and months of watching and beating, and picking with painstaking care.

Latterly establishments have been opened in leading cities to kill moths. First it was done by removing the upholstery from the wood and thoroughly baking it. More recently liquid preparations have been used. But the necessity of transporting furniture to these establishments, and the large expense, have been serious obstacles. Some parties advertised to sell a moth-killing secret for a certain sum of money.

We are happy in being able to announce to the readers of the *American Agriculturist* an easy, simple process that we have tried the past season, with what appears to be a complete success. (We mentioned it to a furniture dealer and repairer to-day, and he said he had practiced it for some time, and that it was a sort of "trade secret."

*The Process*—A set of furniture that seemed to be alive with the larva, from the month it came new, and from which hundreds of these pests had been picked and brushed, was set into a room by itself. Three gallons of benzine were purchased at thirty cents a gallon, retail. Using a small watering-pot with a fine rose sprinkler, the whole upholstery was saturated through and through with the benzine.

*Result.*—Every moth, larva, and egg was killed. The benzine dried out in a few hours and its entire odor disappeared in three or four days. Not the slightest harm happened to the varnish, or wood, or fabrics, or hair stuffing. That was months ago, and not a single moth has since appeared. The carpets were also well sprinkled all round the sides of the room with equally good effect.

For furs, flannels, indeed all woolen articles containing moths, benzine is most valuable. Put them in a box, sprinkle them with benzine, close the box tightly and in a day or two the pests will be exterminated, and the benzine will evaporate on opening.

*Caution.*—Benzine, in fluid or vapor form, is very inflammable, therefore, when using it, have no fire or burning light in the room—not even a match on the floor to be trod on. With this precaution it is safe. With the windows open its odor even will soon disappear. [An outbuilding will be a much safer place for the process. It must be at a safe distance from all fires—say 50 feet or more. ED. BISTOURY.]

---

DANGERS OF MOULDY BREAD.—A singular case of poisoning from eating a pudding made in part of mouldy bread is reported in the *Sanatory Record.* The main facts of the case may be briefly stated as follows: The principal material of the pudding consisted of scraps of bread left from making toast and sandwiches, and they had been about three weeks accumulating. To these scraps were added milk, eggs, sugar, currants, and nutmeg. The whole was baked in a very slow oven, and was subsequently eaten by the cook, the proprietor of the eating-house in which it was prepared, the children of the proprietor and two other persons. All of these became violently ill, with symptoms of irritant poisoning. One of the children (aged three years) and one of the adults died. The autopsy of the body of the child caused the medical men to suspect poisoning, and accordingly the viscera, together with the remnant of the pudding, the materials used in making it, the matter vomited, etc., were sent to a chemical analysist, Mr. Alfred Allen, for examination. He made tests for several poisons, but without positive result. A puppy was fed with the pudding for two days without any poisonous effect. He was then led to look for ergot in the pudding, and was soon startled to find unquestionable evidence of its presence, as far as the chemical reactions went, though he was unable, with the aid of the microscope, to detect any actual ergot. From these facts Mr. Allen infers that the reactions hitherto supposed to be peculiar to ergot, are common to otner poisonous fungi. —*Popular Science Nonthly.*

---

DELICATE TEST FOR WATER.—What is particularly wanted at the present day, and what has not yet been discovered, is a qualitive test which will at once determine whether or not a water is fit for dietetic purposes, and the introduction of such a reagent is the object of a paper by Mr. W. C. Stables in the *Pharmaceutical Journal.* The well known permanganate process is practically a failure, owing to the fact that potassic permanganate does not possess the power of oxidizing albuminoid matter; free ammonia is infallibly detected, while all the important "albuminoid" substances escape untouched. Convinced that potassic permanganate is the base of a very sensitive and delicate test, and that it only requires a little modification to develop it, Mr. Stables began experiments with a view of finding a reagent that would act upon the nitrogenous matter, and bring it under the influence of the potassic permanganate. For this purpose he found that potassic hydrate could not be excelled; and that 4 parts of this, with 1 part of potassic permanganate and 160 parts of freshly distilled water, made the best solution. With such a solution he has made various comparative experiments. One minim, placed in a tube of distilled water, remains of a beautiful pink hue for several days, but the minutest trace of egg albumen in the same quantity of water will be infallibly detected. He states that he has now used this test for some time with most constant results; that is, that if, on the addition of a minim of this solution, the water in a few hours gives a brownish precipitate with loss of color, he has invariably found such water to contain an abnormal proportion of organic matter, so much so as to be injurious to health.

---

TO KEEP BEDS FREE OF BUGS.—I see no end of receipts for cleansing beds, and, as a hotel keeper, have, perhaps, paid over $300 for various nostrums. My plan is, duriug the month of March, to have all my beds taken to pieces, to scrub all the joints and ends with water and soap, and then to use any hard varnish for the ends, slats, etc. I know of no other method as easy or as thorough. I have used this in Georgia and Florida where vermin abound.—*Cor. Druggists' Circular.*

